# The R.M.O. and outside Emergency Calls

**Published:** 1909-01-09

**Authors:** 


### Resident Medical Officers' Department.

### THE R.M.O. AND OUTSIDE EMERGENCY CALLS.

A CORRESPONDENT writes as follows: The communication published under the above heading in THE HOSPITAL on December 12 raises a point which is of considerable moment, not only on its own strict merits, but also because of the general principles involved. Speaking as one who has more than two years' experience as house surgeon and physician in three large hospitals, the writer distinctly inclines to the opinion that an emergency call from without is one that no qualified house officer is justified in neglecting, except only in circumstances such as those detailed, when an emergency equally grave requires his presence within the walls of his own institution.

In large hospitals this latter contingency is unlikely to arise, because if the resident on duty does desert his post temporarily in response to some such urgent call from outside, there is practically always a colleague who can be prevailed on—by interview, message, or telephone—to assume responsibility for the inmates until the return of the departing officer. A jury would quite rightly censure such a house surgeon if it were put to them that a life had been lost, or possibly lost, through his refusal in such circumstances to leave his institution. Another plan at a large hospital would be for the man on duty to get one of his colleagues to answer the call. This would hardly work in practice, as the colleague might be unbooted, or asleep in bed, or otherwise engaged. It is one thing to leave a man on duty to answer possible emergencies in the hospital, and another to call upon him at a moment's notice to fare forth into the streets. Besides valuable time might thus be wasted.

There is, no doubt, something to be said in mitigation of extreme views when the case is that of a small hospital with only one resident; or only one resident actually on the premises at the time. But it is difficult to see how the possibility of a hypothetical emergency arising in the hospital can excuse a man for refusing to attend an actual one outside; the latter is not likely to be far distant, otherwise the house officer will not be the nearest medical man available. To argue the contrary is to invite the corollary that a single-handed R.M.O. must never leave the hospital in which he lives under any circumstances.

To come now to the rules, written and unwritten, which bind or are supposed to bind the actions of hospital medical officers. This is a very large subject indeed, parts only of which can here be discussed. The opinion may be expressed at once that no rule of the governing body of a hospital need be regarded by the R.M.O. if its provisions are obviously contrary to public policy and common sense. This statement may seem supererogatory, but it is a fact that in numerous hospitals rules exist, often very antique ones and, though quite obsolete, unrepealed, the observance of which would produce chaos and confusion at once. It is probable that in many instances these rules are retained merely to afford a convenient handle for getting rid of a resident officer who is tactless or stupid: and from that point of view there may be something to be said in their favour. But the house officer who has the confidence of his medical staff need never fear the consequence of breaking a rule so foolish as that which prohibits his attending a sudden case of dangerous illness or accident outside. In one case a few days ago a man at an eating house almost next door to a hospital contrived to get a bone into his larynx: the house surgeon on duty was summoned, did tracheotomy with a pen-knife on the spot, and saved the man's life. He then asked and obtained a substantial fee for this, and quite rightly so. When the hospital board heard of the matter they tried to make him return the fee, on the ground that by the hospital rules the acceptance of fees was forbidden. However, the attendance of private patients was also forbidden, and the house surgeon who had in the interests of a fellow creature broken one rule declined to be bound by the other, and kept his well earned fee. The ordinary principles of humanity cannot be overridden by hospital rules, and therefore the opinion that "should there be a written rule, he must obey it and refuse to go" is one against which a protest must be entered.

Next arises the question of what to do if, having answered an emergency call from outside, the hospital resident arrives first at the scene of calamity and renders assistance before the arrival of some neighbouring practitioner, also summoned. If the patient is able and willing to pay for private treatment at home, the R.M.O. would be well advised to hand over the case to the practitioner; otherwise to consult with the latter with a view to admitting the patient to hospital. In the latter case no fee can be charged; in the former the procedure in this respect must be determined partly by the nature of the assistance rendered. Thus, if the R.M.O. is detained some time, and especially if he renders highly valuable service, as, for instance, in the case already quoted, he ought certainly to get a fee; for he is not acting in the capacity of hospital officer, but in that of citizen and qualified medical man. Of course if a rule to the contrary effect exists it must be taken into consideration whether the fee is worth the possibility of trouble with the board; all that can be said is that, morally, there can be no stigma attached to the violation of this very common rule in these circumstances.

The fact is that the system of control of medical staff, resident and non-resident, by laymen completely ignorant of the conditions of the work, and nearly always of advanced age, only too often leads to impossible situations. An example in point may be quoted; it occurred only a few weeks ago. A titled subscriber to a hospital sent in a servant to undergo an operation. An hour or two after its performance he telephoned to the hospital to ask how the patient was doing, and owing to the pressure of work on a busy operating afternoon, was kept waiting a quarter of an hour for a reply, which was transmitted over the telephone by the porter. A complaint of this delay was immediately sent to the board, which, without consulting the medical staff or the resident, promptly made a rule that all telephone messages about patients must be answered by the house surgeon in person. Next day this official was engaged in plugging a perinæum for severe hæmorrhage when the porter appeared in the ward, requesting his attendance at the telephone, in accordance with the new rule. Naturally he refused to go, and was duly reported to the board by the Secretary. After hearing the house surgeon the board rescinded its rule. The moral is that the R.M.O. who has reason and the probable support of public opinion and of the medical staff on his side need not be afraid of breaking indefensible rules, of which the commonest is that which forbids him to attend patients otherwhere than within the hospital walls. The rule is almost certainly contrary to public policy in dealing with emergency cases, and cannot be enforced; but it should, of course, be obeyed in other circumstances.

